# High‐mobility group box 1 emerges as a therapeutic target for asthma

**DOI:** 10.1002/iid3.1124

**Published:** 2023-12-22

**Authors:** Qianni Yang, Min Li, Yunjiao Hou, Huilin He, Shibo Sun

**Affiliations:** ^1^ Department of Pulmonary and Critical Care Medicine First Affiliated Hospital, Kunming Medical University Kunming China; ^2^ 2021 Class 2 of Anesthesiology Kunming Medical University Kunming China

**Keywords:** asthma, autophagy, HMGB1, inflammation

## Abstract

High‐mobility group box 1 (HMGB1) is a highly conserved nonhistone nuclear protein found in the calf thymus and participates in a variety of intracellular processes such as DNA transcription, replication and repair. In the cytoplasm, HMGB1 promotes mitochondrial autophagy and is involved in in cellular stress response. Once released into the extracellular, HMGB1 becomes an inflammatory factor that triggers inflammatory responses and a variety of immune responses. In addition, HMGB1 binding with the corresponding receptor can activate the downstream substrate to carry out several biological effects. Meanwhile, HMGB1 is involved in various signaling pathways, such as the HMGB1/RAGE pathway, HMGB1/NF‐κB pathway, and HMGB1/JAK/STAT pathway, which ultimately promote inflammation. Moreover, HMGB1 may be involved in the pathogenesis of asthma by regulating downstream signaling pathways through corresponding receptors and mediates a number of signaling pathways in asthma, such as HMGB1/TLR4/NF‐κB, HMGB1/RAGE, HMGB1/TGF‐β, and so forth. Accordingly, HMGB1 emerges as a therapeutic target for asthma.

## Graphical abstract

High‐mobility group box 1 (HMGB1) is a highly conserved non‐histone nuclear protein found in the calf thymus and participates in a variety of intracellular processes such as DNA transcription, replication, and repair. HMGB1 emerges as a therapeutic target for asthma.

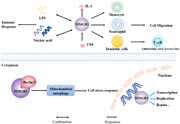

## 1 INTRODUCTION

Asthma is a common respiratory disease that is estimated to affect about 300 million people worldwide, and the number of asthma is expected to increase significantly in the next 20 years due to changes in environmental factors such as air pollution.^1^ Asthma is characterized by airway hyperresponsiveness, repeated airway inflammation, and reversible airway obstruction that causes dyspnea in patients.^2^ HMGB1 has a dual effect on the immune function of CD4 + T lymphocytes. Low dose of HMGB1 has no effect on the proliferative activity of CD4 + T lymphocytes, but production of Th1 cytokine increases. On the contrary, high‐dose HMGB1 inhibits the proliferative response of CD4 + T lymphocytes to induce Th2 polarization.^3^ It is suggested that mutual regulation between Th1/Th2 and Th17/Treg plays an important role in asthma. However, the Th1/Th2 and Th17/Treg are different because the former is related to allergic asthma while the latter to nonallergic asthma.^4^, ^5^, ^6^ However, the underlying mechanisms are still poorly understood. The classic high mobility group protein (HMG) proteins fall into three categories: high mobility group protein B (HMGB), high mobility group protein A (HMGA), and high mobility group protein G (HMGN).^7^, ^8^, ^9^ Studies have shown that HMGA and HMGN are involved in the inflammatory response in lung cancer^10^, ^11^ and HMGB includes four types of proteins which are named HMGB1, 2, 3, and 4.^12^ Among these proteins, HMGB2 and 3 are mainly related to the occurrence of various cancers.^13^, ^14^ While HMGB4 is mainly related to the regulation of neuronal differentiation and expression.^15^ As a late‐stage inflammatory agent, HMGB1 is a highly conserved nonhistone nuclear protein found in the calf thymus and is involved in various inflammatory responses and autoimmune processes.^16^ Increasing studies have shown that HMGB1 plays a role in different acute and chronic airway inflammation and plays an important role in the occurrence and development of respiratory diseases such as asthma.^17^, ^18^, ^19^ This article reviews the role and mechanism of HMGB1 in asthma.

## 2 STRUCTURE AND FUNCTION OF HMGB1

**Figure 1:**
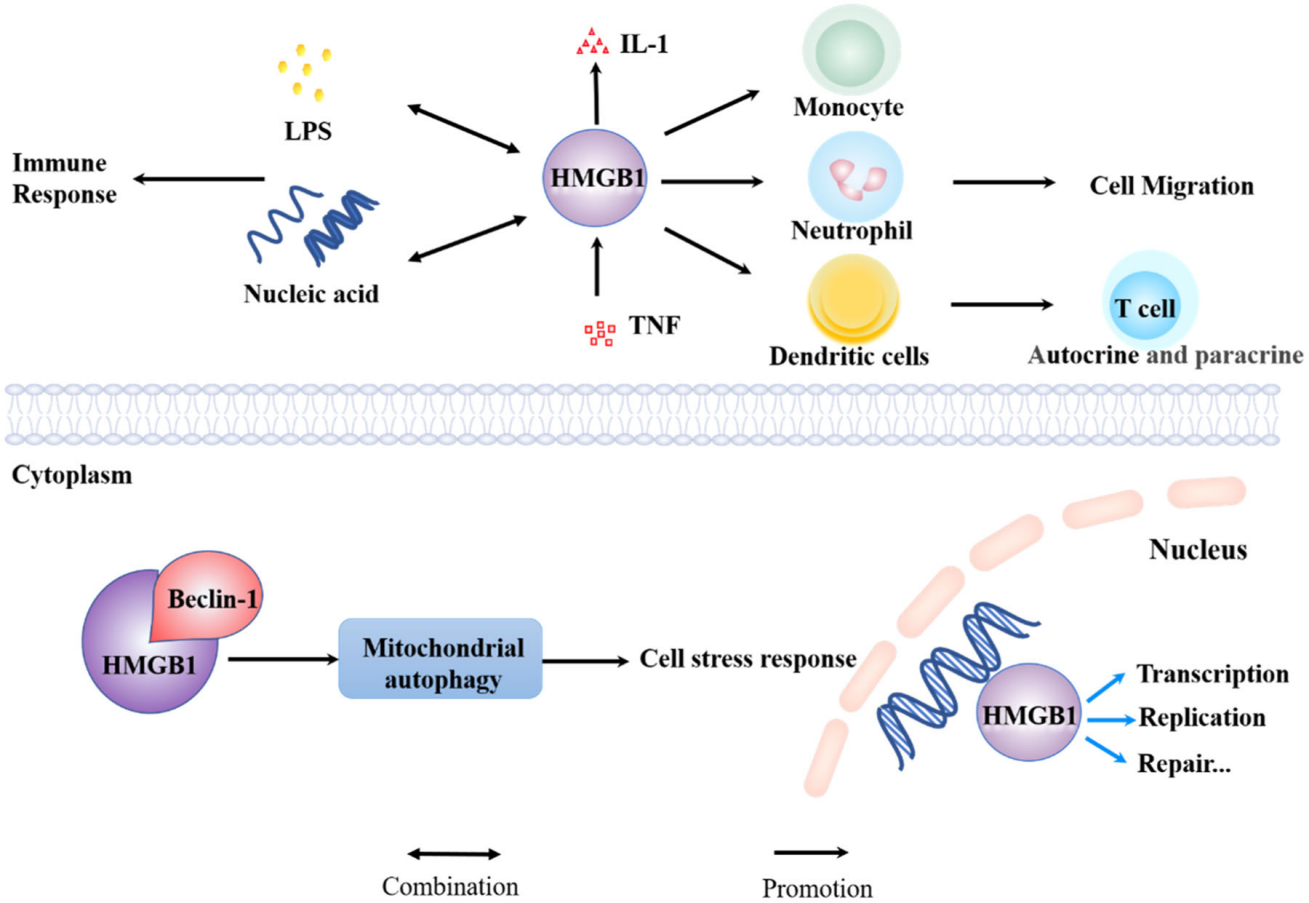
HMGB1 inside and outside the cell presents different functions. HMGB1, high‐mobility group box 1; IL‐1, interleukin‐1; LPS, lipopolysaccharide; TNF‐α, tumor necrosis factor.

HMGB1 consists of three distinct domains: two proximal homologous DNA binding sequences (Box‐A and Box‐B) and a negatively charged C‐terminal.^20^ The Box‐A region has anti‐inflammatory and therapeutic potential, while the Box‐B region contains the molecular of pro‐inflammatory cytokine functions.^21^ HMGB1 contains two nuclear localization sequences recognized by the intracellular transport complex, which determine the location of HMGB1 in the nucleus or cytoplasm under physiological conditions.^22^, ^23^ Intracellular HMGB1 is mainly located in the nucleus. As a kind of nuclear protein that bind various types of DNA with high affinity, HMGB1 maintains the stability of nucleosomes and participates in key biological reactions such as DNA transcription, replication, repair, recombination and maintenance of telomere stability.^24^, ^25^ Cytoplasmic HMGB1 binds Beclin‐1 protein, which promotes mitochondrial autophagy and participate in cell stress response.^26^ Once released outside the cell, HMGB1 becomes an inflammatory factor that triggers inflammatory responses and participates in a variety of immune responses.^27^ For example, (1) HMGB1 acts as a pro‐inflammatory mediator in infection or aseptic tissue injury, activating the release of tumor necrosis factor‐alpha (TNF‐α) and interleukin‐1 (IL‐1) in a dose‐dependent manner.^28^, ^29^, ^30^ In addition, these inflammatory factors also promote the synthesis and release of HMGB1^31^, ^32^; (2) HMGB1 can promote the migration of monocytes, dendritic cells and neutrophils^33^; (3) It forms the complex with almost all kinds of nucleic acid and endotoxin lipopolysaccharide (LPS) to enhance the immune response^34^, ^35^; (4) In adaptive immune response, HMGB1 induce dendritic cell maturation and promote T lymphocyte proliferation in an autocrine/paracrine manner.^36^, ^37^ Inhibition of HMGB1 acetylation can reduce neuroinflammatory response and restore cell redox homeostasis.^38^ Accordingly, HMGB1 proteins distributed in different locations inside and outside the cell present different functions.^39^ Meanwhile, it is reported that HMGB1 promotes and induces asthma through immune system, secretion of enzyme‐promoting inflammatory factors, and signal transduction.^40^, ^41^, ^42^ More importantly, the administration of HMGB1 inhibitor after severe infection can significantly reduce the relevant inflammatory response after changing its mRNA expression.^19^, ^43^ However, the role of HMGB1 protein in human remains to be further elucidated (Figure 1).

## 3 PATHWAYS RELATED TO HMGB1

### 3.1 RAGE pathway

Advanced glycation end product (RAGE) is a pattern recognition receptor involved in the recognition of endogenous molecules released due to cell death or tissue damage in infection, physiological stress, or chronic inflammation.^44^, ^45^ It is shown that sRAGE (the soluble form of RAGE) prevents Th17‐mediated airway inflammation in neutrophil asthma at least in part by blocking HMGB1/RAGE signaling in dendritic cell (DC).^46^ Additionally, HMGB1 induces a cascade of signaling by activating the RAGE through increasing secretion of HMGB1 and the expression of receptors that interact with HMGB1, mediating the amplification of inflammation and angiogenesis and promoting inflammation.^47^

Recently, It is suggested that the cytoplasmic translocation of HMGB1 in microglia cells is increased under chronic stress and the expression of its receptor RAGE is upregulated, which affects the inflammatory response by damaging microglia mitochondria autophagy and initiating the production of pro‐inflammatory cytokines (PICs).^48^ Similarly, in spinal cord injury (SCI) mice, the HMGB1/RAGE pathway is involved in the major pro‐inflammatory macrophage/microglia‐mediated pro‐inflammatory response. Inhibition of HMGB1 or RAGE can effectively reduce the number of inflammatory macrophages/microglia before injury and increase the number of anti‐inflammatory cells. Inhibition of this pathway has neuroprotective effect on SCI.^49^

### 3.2 NF‐κB pathway

The NF‐κB pathway is considered as a typical pro‐inflammatory signaling pathway.^50^ NF‐κB is also an important regulator of immunity, stress response, apoptosis, and differentiation.^51^ It also plays an important role in other processes, including development, cell growth and survival, and proliferation.^52^ Inhibition of the NF‐κB pathway reduces the production of Th2 and Th17 cytokines, which is potential therapeutic targets to asthma.^53^

Activation of NF‐κB is widespread in tumors and is primarily driven by inflammatory cytokines in the tumor microenvironment.^54^ HMGB1 may be associated with the risk of cancer aggression in prostate cancer. In addition, HMGB1 directly interacts with TNFR1 and promotes the overexpression of MMP‐1, MMP‐3, and MMP‐10 by activating the NF‐κB signaling pathway.^55^, ^56^ It is confirmed that gastric carcinoma cell‐derived exosomes (GC‐Ex) induce increased autophagy response of neutrophils by transporting HMGB1 and interacting with TLR4 to activate the NF‐κB pathway and promote tumor activation.^57^

**Figure 2:**
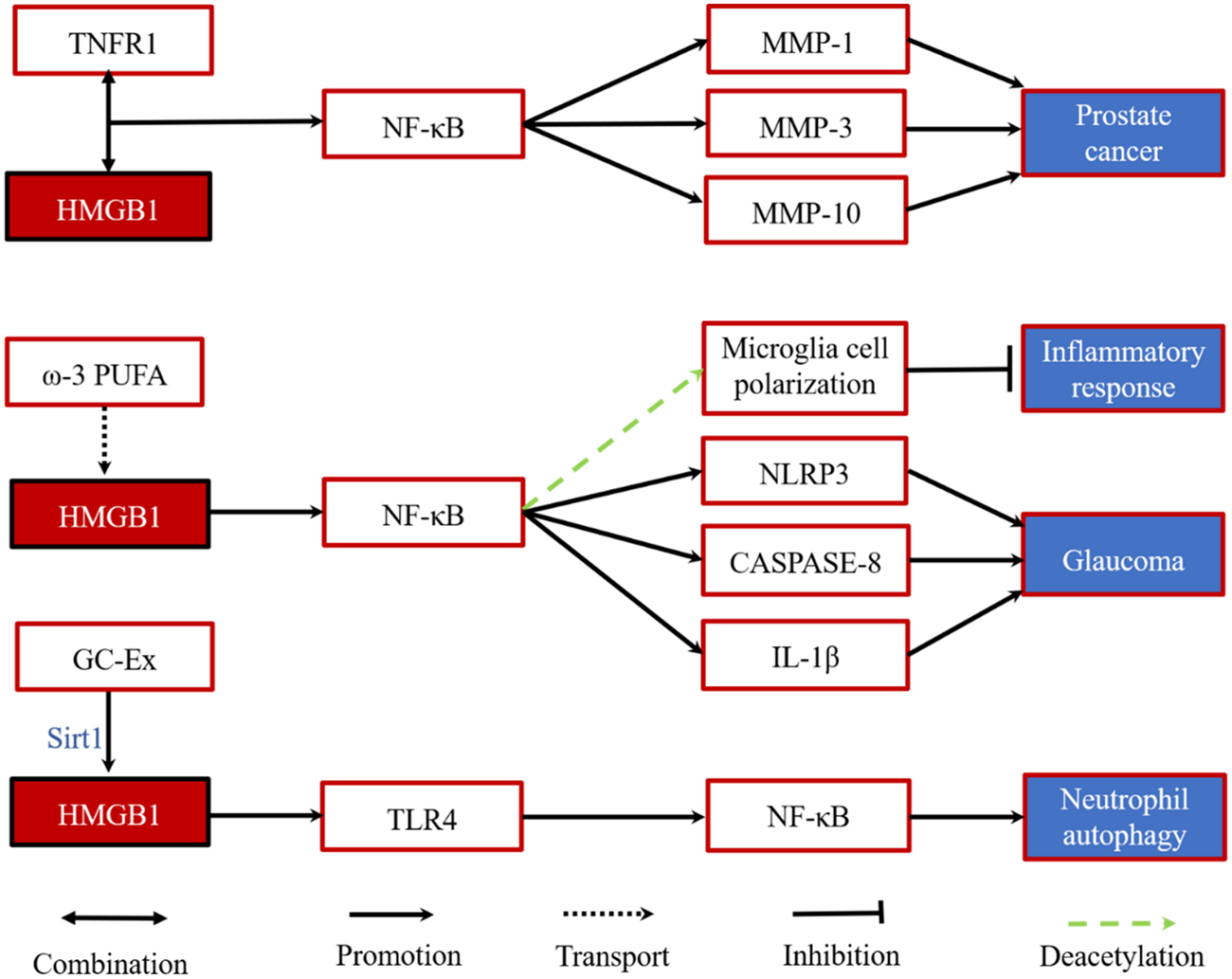
The role of HMGB1 in NF‐κB pathway. GC‐Ex, gastric carcinoma cell‐derived exosomes; HMGB1, high‐mobility group box 1; IL‐1β, interleukin‐1β; ω−3 PUFA, ω−3 polyunsaturated fatty acids; NF‐κB, nuclear factor κB; NLRP3, leucine‐rich repeat‐containing protein 3; TLR4, toll‐like receptor 4.

Related inflammatory responses induced by this pathway, such as ω−3 polyunsaturated fatty acids (omega‐3 PUFA) regulate microglia cell polarization through SIRT1‐mediated HMGB1/NF‐κB pathway deacetylation, thereby reducing inflammatory responses after brain injury to play a neuroprotective role.^58^ In addition, during the development of acute glaucoma, HMGB1 activates the production of typical NLRP3, atypical caspase‐8 inflammatory bodies, and IL‐1β through the NF‐κB pathway in response to acute ocular pressure increase, which can be regarded as a congenital response in the pathogenesis of glaucoma^59^ (Figure 2).

### 3.3 JAK/STAT pathway

JAK1/STAT3 pathway promotes the differentiation of naïve CD4 + T cell into Th17 lymphocytes through phosphorylation, and the proportion of Th17 cells is downregulated with inhibition of JAK1‐STAT3 pathway phosphorylation.^60^ Th17 cells produce cytokines such as IL‐17, which stimulates neutrophil airway inflammation and play a role in nonallergic asthma. Accordingly, the JAK1/STAT3 pathway is associated with nonallergic asthma.^61^

JAK/STAT signaling pathway is a universally expressed signal transduction pathway in cells, which is involved in the signal regulation of various inflammatory mediators, and its continuous activation is closely related to many immune and inflammatory diseases.^62^, ^63^ It is reported that HMGB1 expression is significantly increased in macrophages after LPS stimulation in vitro, which leads to the activation of the JAK/STAT pathway.^64^ JAK‐STAT pathway inhibitor pretreatment can significantly reduce HMGB1 mRNA expression but has no effect on HMGB1 release.^65^ Accordingly, both JAK/STAT pathway and HMGB1 are closely related to inflammatory cytokines such as TNF‐α, IL‐1 and IFN‐γ.^64^, ^66^, ^67^, ^68^

**Figure 3:**
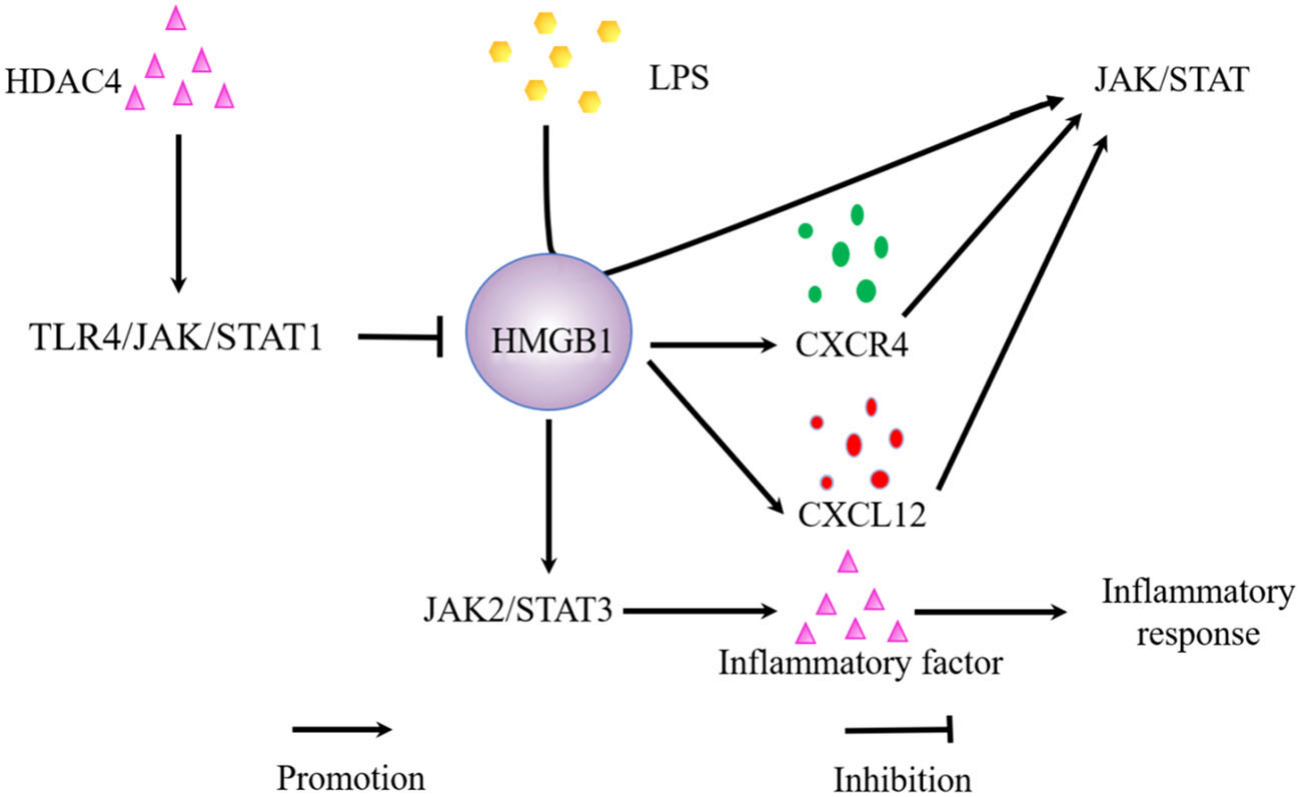
The role of HMGB1 in JAK/STAT pathway. HDAC4, histone deacetylase 4; HMGB1, high‐mobility group box 1; JAK/STAT, Janus kinase/signal transducer and activator of transcription; LPS, lipopolysaccharide.

In sepsis, histone deacetylase 4 (HDAC4) can significantly inhibit the acetylation and secretion of HMGB1 through overexpression of TLR4/JAK/STAT1 pathway, thus inhibiting inflammation.^69^ In addition, HMGB1 in its reduced form binds to CXCL12 via the chemokine receptor CXCR4 in rheumatoid arthritis (RA), and a high concentration of CXCL12/HMGB1 hybrid complex is dependent on the JAK/STAT pathway to maintain the inflammatory response.^70^ In severe acute pancreatitis (SAP), HMGB1 induces the activation of JAK2/STAT3 signaling pathway in rat pancreatic acinus cells to cause the release of a large number of inflammatory cytokine cells, which aggravates the inflammatory response^71^ (Figure 3).

It is reported that resveratrol inhibits the transfer of HMGB1 from nucleus to cytoplasm through JAK/STAT pathway to reduce the release of HMGB1, thus play an anti‐inflammatory role.^72^ Glycyrrhizic acid can inhibit lung cancer cell growth by inhibiting the phosphorylation of JAK2 and STAT3, thereby reducing the activity of the upstream regulator JAK/STAT signaling pathway of HMGB1.^73^ By inhibiting JAK2/STAT3 signaling pathway, curcumin reduces the expression of HMGB1 in brain tissue after cerebral ischemia and reduces the release of TNF‐α and other inflammatory factors after cerebral ischemia, which significantly reduce the inflammatory response after cerebral ischemia.^74^

## 4 THE ROLE AND RELATED PATHWAYS OF HMGB1 IN ASTHMA

In recent years, increasing studies suggest that HMGB1 may cause cell damage and mediate inflammatory response in lung tissue of patients with asthma. It is reported that the level of HMGB1 increases in asthma.^75^ In addition, the signaling pathway involved in HMGB1 plays a key role in inflammation and immune response. It is confirmed that Ping Chuanning Decoction (PCN) reduces the inflammatory response of human airway epithelial cells (16HBE) by inhibiting autophagy mediated by ROS/HMGB1/Beclin‐1. Accordingly, it may be a potential therapeutic target to bronchial asthma.^76^, ^77^, ^78^ The abnormal structure, expression, and activation of HMGB1 may mediate inflammatory response to affect the occurrence and development of asthma. It is reported that HMGB1 levels in sputum of asthmatic patients are significantly increased and positively correlated with asthma severity, obesity, and neutrophil percentage and significantly increased in lung tissue and bronchoalveolar lavage fluid (BALF) of asthmatic rats induced by ovalbumin (OVA) and positively correlated with obesity.^79^, ^80^, ^81^ HMGB1 levels were negatively correlated with lung function in asthmatic patients.^82^, ^83^, ^84^ This means that HMGB1 may be a potential biomarker of asthma severity.^85^ In addition, inhibition of HMGB1 protects human bronchial epithelial cells (HBE) by alleviating TDI‐induced asthma via ROS/AMPK/autophagy pathway.^86^ Meanwhile, HMGB1 is related to allergic asthma through ferroptosis.^87^ In addition, HMGB1 is the substrate of RNF125 which is downregulated in the bronchial epithelium of asthma patients. Increasing the stability of HMGB1 promotes autophagy induced by oxidative stress to improve asthma.^88^ Moreover, HMGB1, as a pro‐inflammatory factor, is secreted by bronchial epithelial cells and drives the release of inflammatory cytokines. While inhibition of HMGB1 reduces the lung inflammatory cell infiltration, goblet cell proliferation, and airway mucus production to improve asthma.^89^ Accordingly, HMGB1 plays an important role in asthma.

**Figure 4:**
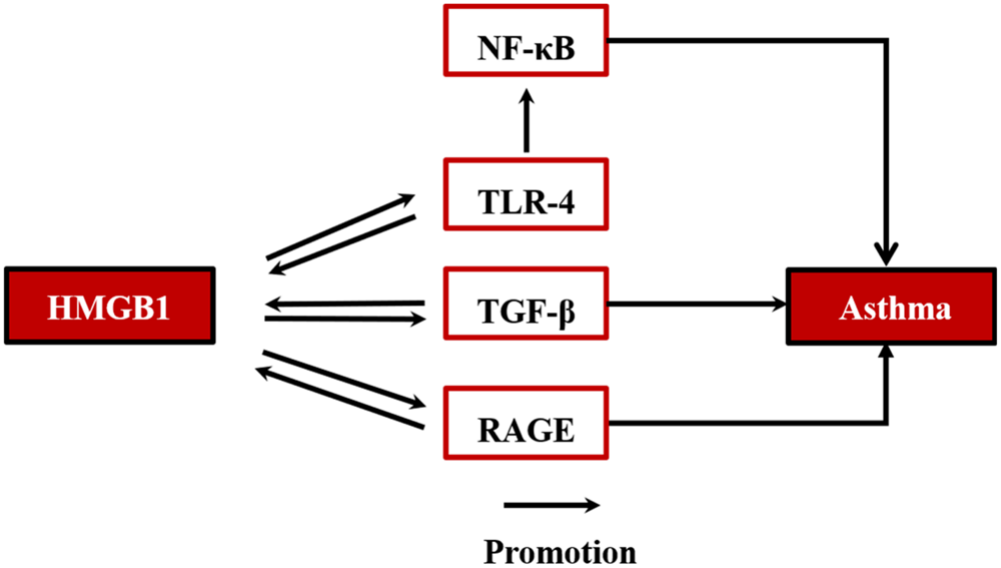
The signaling pathways related to HMGB1 in asthma. HMGB1, high‐mobility group box 1; NF‐κB, nuclear factor κB; RAGE, advanced glycation end products; TGF‐β, transforming growth factor‐β; TLR4, toll‐like receptor 4.

HMGB1/TLR4/NF‐κB, HMGB1/RAGE, JAK/STAT, and HMGB1/TGF‐β play important roles in the occurrence and development of asthma. All of these pathways activate the HMGB1 to result in a range of inflammatory responses and damage of cells^90^, ^91^, ^92^, ^93^ (Figure 4). For example, HMGB1/RAGE signaling synergically enhances TGF‐β1‐induced broncho‐airway remodeling and epithelial‐mesenchymal transformation (EMT) by promoting Th17 cell differentiation and IL‐17 secretion, which is one of the causes of severe steroid‐resistant asthma.^94^

It is suggested NF‐κB and TLR4 are activated to cause the airway inflammation, while administration of anti‐HMGB1 antibodies or ethyl pyruvate (EP) reduces airway inflammation and remodeling in mice asthma models induced by OVA.^95^, ^96^ Accordingly, both HMGB1/NF‐κB and HMGB1/TLR4 signals may be involved in the occurrence and development of asthma.^97^ Although there is evidence that HMGB1 protein plays an important role in the pathogenesis of asthma, the specific expression and distribution of HMGB1 protein in the airway and lungs have not been conclusive.

## 5 CONCLUSIONS

HMGB1 is involved in the occurrence of many diseases and plays an important role in asthma. In addition, the main signaling pathway of HMGB1 involved in asthma is HMGB1/TLR4/NF‐κB and HMGB1/RAGE, while whether the JAK/STAT signaling pathway is involved in HMGB1 still unclear at present. HMGB1 is a therapeutic target of asthma, but more research works are needed to investigate the role of HMGB1 in asthma and its mechanism in future.

## AUTHOR CONTRIBUTIONS

**Qianni Yang**: Formal analysis; investigation; methodology; writing—original draft. **Min Li**: Data curation; funding acquisition; methodology; writing—review & editing. **Yunjiao Hou**: Formal analysis; investigation. **Huilin He**: Formal analysis; investigation.

## CONFLICT OF INTEREST STATEMENT

The authors declare no conflict of interest.

## Data Availability

The data in this article are all included in the content. The data that support the findings of this article are included within this article (Figures 1, 2, 3, 4).
